# Exploring Psychological Distress and Adverse Childhood Experiences Among U.S. Women with Concurrent Reproductive Trauma and Infertility: A Secondary Analysis of a Cross-Sectional Study

**DOI:** 10.1177/26884844251397926

**Published:** 2025-11-20

**Authors:** Alison Swift, Anna Schroeder, Melvin Swanson, Madeline Fernandez-Pineda

**Affiliations:** ^1^ Department of Advanced Nursing Practice and Education, East Carolina University, Greenville, North Carolina, USA.; ^2^ ECU College of Nursing, Greenville, North Carolina, USA.; ^3^ Department of Nursing Science, East Carolina University College of Nursing, Greenville, North Carolina, USA.; ^4^ Department of Nursing Science, East Carolina University, Greenville, North Carolina, USA.

**Keywords:** psychological distress, adverse childhood experiences, reproductive trauma, infertility, pregnancy loss

## Abstract

**Background:**

Adverse childhood experiences (ACEs) are traumatic events that occur during childhood. Previous evidence suggests that pregnancy loss may be associated with ACEs and mental health disorders; however, to our knowledge, no studies have considered these associations in women with concurrent reproductive trauma (CRT), defined as pregnancy loss during infertility.

**Purpose:**

To compare ACEs, stress, anxiety, depression, and post-traumatic stress disorder (PTSD) symptoms among women with CRT and women with infertility only.

**Methods:**

A descriptive cross-sectional study design was used to recruit 99 U.S. women with infertility through convenience and snowball sampling on Facebook, Instagram, and at fertility clinics. A REDCap survey included demographics, ACEs questionnaire, infertility-related stress (COMPI-FPSS), perceived stress (PSS-4), anxiety (GAD-7), depression (PHQ-8), and PTSD (PC-PTSD-5). Statistical analysis using SPSS 28 included descriptive statistics, independent *t*-tests, and chi-square test for independence.

**Results:**

Women with CRT were more likely to experience 2+ ACEs than women with infertility-only (*p* = 0.001), with four ACEs occurring more often. Infertility-only group with 2+ ACEs had significantly higher levels of stress, PTSD, anxiety, and depression, with clinically significant higher depression and PTSD symptoms. CRT group with 2+ ACEs had significantly higher anxiety and depression, with clinically significant higher moderate or severe depression.

**Discussion:**

Although women with CRT and infertility experienced similar levels of psychological distress, having an ACE history worsened their mental health symptoms. ACE history may be associated with reproductive traumas, though further studies are needed. Trauma-informed care should be implemented for women with ACEs, infertility, and CRT.

## Introduction

Reproductive traumas, such as infertility and pregnancy loss, are traumatic reproductive events that cause significant psychological distress.^1^ In the United States, one in five women, or individuals assigned female sex at birth, experience infertility, characterized by the inability to conceive or sustain a pregnancy after 12 months of unprotected sexual intercourse.^2^ The experience of infertility is coupled with infertility-related stress,^3^ depression, post-traumatic stress disorder (PTSD) symptoms,^4^ and lower quality of life.^5^ Fertility treatments, such as medications to induce ovulation or more advanced treatments like *in vitro* fertilization, are used to help women achieve pregnancy but can be an additional source of stress for women.^6^ Women who undergo treatment in the United States often express concerns related to financial and occupational-related stress, which may be due to the out-of-pocket expenses to pay for fertility treatments and missed work time due to treatments that require specific interventions during the menstrual cycle.^3^

In addition, pregnancy loss during fertility treatments, also called concurrent reproductive trauma (CRT), can increase infertility-related stress, decrease quality of life,^7^ and cause significant anxiety and depression in women with infertility who later become pregnant after fertility treatments.^8^ Pregnancy loss most often occurs prior to 13 weeks of gestation, with estimates around 13.5% of all clinically recognized pregnancies;^9^ however, the incidence of pregnancy loss during fertility treatments is unknown. Pregnancy loss is associated with psychological distress, including anxiety, stress, and depressive symptoms,^10^ and an increased risk of suicide in women.^11^ Brigance et al.^12^ reported that women who experienced infertility or pregnancy loss had significantly more trauma symptoms than those who did not experience reproductive traumas. Thus, the concurrent experience of pregnancy loss during infertility may exacerbate the psychological distress and trauma symptoms of women who are already experiencing significant stress from being unable to conceive.

Furthermore, previous traumatic experiences, whether actual or threatened, are associated with mental health disorders.^13,14^ In particular, childhood traumatic experiences are associated with later adult mental health disorders,^15^ including psychiatric disorders and depressive symptoms.^16^ Adverse childhood experiences (ACEs) are traumatic events that occur during childhood, including forms of physical, emotional, or sexual abuse; physical or emotional neglect; divorce; parental death; parental incarceration; witnessing violence or death; and experiences with substance use or mental illness in the home.^17^ ACEs have also been found to have a dose-response effect, where each additional ACE worsens mental health outcomes. Daníelsdóttir et al.^16^ conducted a cohort study of over 25,000 individuals and found that each additional ACE experienced was associated with 52% greater odds of a psychiatric disorder diagnosis.

Not only do ACEs affect later adult mental health, but ACEs are also associated with adult chronic diseases, including coronary artery disease, heart attack, stroke, diabetes, cancer, chronic bronchitis, emphysema, substance use, and depression.^18,19^ However, there is limited evidence of associations between ACEs and later adult reproductive diseases and reproductive traumas. Previous evidence suggests that pregnancy loss may be associated with ACEs,^20^ and each additional ACE experienced increased the risk of pregnancy loss by 12%.^21^ Kerkar et al.^22^ found that women had between 1.7- and 1.9-fold higher odds of pregnancy loss if they had experienced at least four ACEs compared with women with no ACEs. To our knowledge, no studies have considered associations between ACEs and pregnancy loss in the context of women with infertility or have considered relationships between ACEs and mental health disorders in women with reproductive traumas. Therefore, the purpose of this study was to compare ACEs and psychological distress (infertility-related stress, perceived stress, anxiety, depression, and PTSD scores) among women with CRT and women with infertility alone.

## Materials and Methods

### Study design

This study is a secondary analysis of a parent study using a descriptive cross-sectional study design to explore ACEs, chronic stress biomarkers, and psychological distress among United States (U.S.) women with infertility and U.S. mothers. This study was approved by the University Medical Institutional Review Board at East Carolina University. Informed consent was obtained from participants prior to the start of the online survey by participants selecting “agree” to participate in the study.

### Participant sample/setting

Participants were recruited between November 2023 and May 2024 through convenience sampling. Administrators from infertility-related support groups on Facebook and Instagram influencers were asked to share the study information and a survey link with their followers. Participants were also recruited through snowball sampling when Facebook users or Instagram users shared the study information or survey link with other social media users. In addition, participants were also recruited through a posted flyer at three health care and fertility clinics in Florida and North Carolina. Inclusion criteria for participants included those who were biologically female sex, had an infertility diagnosis and were undergoing fertility treatments at the time of the study, resided in the U.S., were between the ages of 18 and 49, and were able to read English or Spanish. Exclusion criteria included biologically male sex, living outside of the U.S., being younger than 18 years or older than 49 years, and being unable to read English or Spanish. A power analysis was conducted to determine the sample size. Using a power of 0.80, *α* = 0.05, and a moderate effect size, the estimated sample size is *n* = 40 for each group.

### Data collection

Survey data were collected on REDCap, a Health Insurance Portability and Accountability Act (HIPAA) approved software application to develop and store online survey data.^23^ Administrators from infertility-related Facebook groups and Instagram influencers/pages were contacted through the direct message on each of the social media platforms and were asked to share the survey link and study information or permission for a research team member to post the link and information in the group. Facebook and Instagram users could also share the study link and information with other social media users. Administrators from health care and fertility clinics in Florida and North Carolina were also contacted and asked to post a study flyer that contained study information and a Quick-Response (QR) code that would link to the survey. The survey was available in both English and Spanish and included an electronic consent, screening questions, infertility-related stress, perceived stress scale, anxiety, depression, PTSD, quality of life, ACE scores, reproductive history, and demographic information.

### Measures

#### Copenhagen Multi-Center Psychosocial Infertility Fertility Problem Scale Score

The Copenhagen Multi-Center Psychosocial Infertility Fertility Problem Scale Score (COMPI-FPSS) is a nine-item scale to measure personal (three items), social (three items), and marital (three items) infertility-related stress. The scale includes two items on a five-point Likert scale ranging from *strongly disagree* (1) to *strongly agree* (5), and seven items on a four-point Likert scale ranging from *not at all* (1) to *a great deal* (4). Higher scores indicate higher infertility-related stress in each of the subscales.^24,25^ Internal consistency for coefficient alphas for the subscales in this study was 0.49 for personal stress, 0.64 for marital stress, and 0.76 for social stress.

#### Perceived Stress Scale

The Perceived Stress Scale (PSS-4) is a four-item, five-point Likert scale measuring stress within the past 2 weeks. The scale ranges from *never* (0) to *very often* (4). Higher scores indicate higher stress.^26^ The coefficient alpha for this study was 0.73.

#### Generalized Anxiety Disorder Scale

The Generalized Anxiety Disorder Scale (GAD-7) is a seven-item, four-point Likert scale, ranging from *not at all* (0) to *nearly every day* (3). The scale measures anxiety symptoms over the past 2 weeks. Higher scores indicate more anxiety-related symptoms^27^ with scores higher than 10 indicating clinical anxiety. Cronbach’s alpha for internal consistency was 0.88 in our study.

#### Patient Health Questionnaire

The Patient Health Questionnaire (PHQ-8) is an eight-item, four-point Likert scale questionnaire measuring depression symptoms over the past two weeks. The questionnaire ranges from *not at all* (0) to *nearly every day* (3). Higher scores indicate more depression-related symptoms,^28^ with scores higher than 10 indicating clinical depression. In our study, the coefficient alpha was 0.88.

#### Primary Care PTSD Screen for DSM-5

The Primary Care PTSD Screen for DSM-5 (PC-PTSD-5) is a five-item screening tool measuring PTSD symptoms over the past 2 weeks. The screening includes a *yes* or *no* answer choice for all five questions. A higher number of *yes* responses indicates more PTSD-related symptoms,^29^ with a score of 3 or more indicating probable PTSD symptoms. The coefficient alpha was 0.73 in our study.

#### Adverse childhood experience

The ACEs Questionnaire is a 10-item questionnaire of ACEs experienced prior to one’s 18^th^ birthday. The questionnaire includes a *yes* or *no* response choice for all 10 ACE items, with a *yes* response indicating the participant experienced that ACE prior to their 18^th^ birthday, and a *no* response indicating the participant did not experience that ACE item. A higher number of *yes* responses indicates more ACEs experienced during childhood.^30^

### Data analysis

Statistical analysis was conducted using SPSS 28. Descriptive statistics were used to describe demographic and reproductive characteristics. Chi-square tests for independence and independent *t*-tests were used to examine differences in infertility-related stress, perceived stress, anxiety, depression, PTSD, and ACE scores among women with CRT and women with infertility alone.

## Results

Of the 99 participants in this study, 59 (60%) experienced CRT, and 40 (40%) had infertility-only. Most participants in both the CRT and infertility-only groups were non-Hispanic or Latino, Caucasian/White, and had an education level equal to or above a baccalaureate degree. Most of the participants were employed full-time, and the household yearly income was about evenly distributed between $51,000–$100,00, $101,000–$150,000, and >$150,000. Participants tended to live in an urban area compared with a rural or suburban area. Most participants had primary infertility and had partial or no fertility insurance coverage (Table 1).

**Table 1.** Demographic Characteristics of Study Participants (*N* = 99)

	CRT		Infertility only	
Characteristic	*n*	%	*n*	%
Ethnicity				
Non-Hispanic or Latino	52	88.1	37	92.5
Hispanic or Latino	6	10.2	3	7.5
Other	1	1.7	0	0.0
Race				
African American/Black	3	5.1	4	10.0
Asian	1	1.7	1	2.5
Caucasian/White	56	94.9	36	90.0
Native Hawaiian or Pacific Islander	1	1.7	0	0.0
Multi-Race or Mixed Race	3	5.1	1	2.5
Education				
Some college or less	5	8.5	2	5.0
Technical/associate degree	1	1.7	3	7.5
Some 4-year college	1	1.7	3	7.5
Baccalaureate degree or higher	52	88.2	32	80.0
Marital status				
Married	54	91.5	37	92.5
Committed relationship	2	3.4	2	5
Single or never married	3	5.1	1	2.5
Household yearly income (U.S. dollars)				
<50,000	6	10.2	3	7.5
51,000–100,000	15	25.5	12	30.0
101,000–150,000	19	32.2	12	30.0
>150,000	19	32.2	13	32.5
Employment status				
Unemployed	9	15.3	2	5.0
Part-time employment	5	8.5	8	20.0
Full-time employment	45	76.3	30	75.0
Area living in				
Rural	10	16.9	7	17.5
Urban	28	47.5	10	25.0
Suburban	21	35.6	23	57.5
Fertility insurance coverage				
Full coverage	6	10.2	1	2.5
Partial coverage	28	47.5	19	47.5
No coverage	25	42.4	20	50.0
Infertility type				
Primary	41	69.5	27	67.5
Secondary	14	23.7	11	27.5
Unknown/unsure	4	6.8	2	5.0

Note: CRT, concurrent reproductive trauma.

We compared the frequency of responses of each ACE item between the CRT group and infertility-only group using the Chi-square test for independence (Table 2). Compared with the infertility-only group, the CRT group reported higher ACEs on each item except ACE item 10, which asked participants if they had experienced sexual abuse as a child. Four of the 10 ACE items were selected significantly more in the CRT group, which included ACE item 1 (neglected, underfed, unprotected), ACE item 4 (substance use in the household), ACE item 6 (household member incarcerated), and ACE item 7 (verbal abuse).

**Table 2.** Adverse Childhood Experience Scale Item Differences Between Concurrent Reproductive Trauma (*n* = 59) and Infertility Only (*n* = 40) Groups

	CRT		Infertility only		**χ**^2^ (1)	*p*
Variable	*n*	%	*n*	%		
ACEs items						
1 Neglected, underfed, unprotected	11	18.6	2	5.0	3.89	0.049
2 Deceased or absent parent	21	35.6	10	25.0	1.24	0.265
3 Depressed or suicidal household	26	44.1	15	37.5	0.42	0.515
4 Substance-use household	25	42.4	8	20.0	5.37	0.020
5 Domestic violence at home	12	20.3	4	10.0	1.88	0.170
6 Household member incarcerated	10	16.9	1	2.5	5.04	0.025
7 Verbal abuse at home	36	61.0	14	35.0	6.45	0.011
8 Physical abuse at home	14	23.7	6	15.0	1.13	0.288
9 Unloved or undervalued at home	14	23.7	4	10.0	3.02	0.082
10 Sexual abuse	12	20.3	10	25.0	0.30	0.584

Note: ACE, adverse childhood experience; CRT, concurrent reproductive trauma.

Table 3 presents the mean psychological distress scores between the CRT group and the infertility-only group. Mean psychological distress scores were higher in the CRT group compared with the infertility-only group, except for the PHQ8 score; however, they were not statistically significant.

**Table 3.** Mental Health Analysis of Total Participants (*n* = 99), Concurrent Reproductive Trauma (*n* = 59), and Infertility Only (*n* = 40) Groups

	Total (*n* = 99)		CRT (*n* = 59)		Infertility only (*n* = 40)			
Variable	*M*	SD	*M*	SD	*M*	SD	*t* (97)	*p*
Mental health scales								
FPSS—personal stress	11.53	1.40	11.69	1.37	11.28	1.43	1.47	0.145
FPSS—marital stress	8.42	2.28	8.53	2.26	8.27	2.32	0.53	0.594
FPSS—social stress	6.75	2.35	6.95	2.26	6.45	2.49	1.03	0.303
Perceived stress	8.44	2.54	8.39	2.62	8.53	2.44	0.26	0.796
PTSD	3.14	1.62	3.31	1.51	2.90	1.75	1.23	0.223
GAD	10.98	5.36	11.58	5.02	10.10	5.78	1.35	0.180
PHQ8	9.66	5.72	9.56	5.61	9.80	5.95	0.20	0.838

Note: CRT, concurrent reproductive trauma; FPSS, Fertility Problem Scale Score; GAD, Generalized Anxiety Disorder Scale; PHQ8, Patient Health Questionnaire; PTSD, post-traumatic stress disorder.

We used independent *t*-tests to compare psychological distress scores between 0–1 ACEs and 2+ ACEs among CRT and infertility-only groups (Table 4). Of the 59 participants who experienced CRT, 40 (68%) had 2 or more ACEs, whereas 14 (35%) of the 40 participants with infertility-only had 2 or more ACEs (χ^2^ = 10.34, *p* = 0.001). The CRT group had higher means in each of the mental health scales when experiencing 2+ ACEs, compared with mental health scores among those with CRT experiencing 0–1 ACEs, with statistically significant differences between the GAD-7 and PHQ-8 mean scores. In the infertility-only group, participants who experienced 2+ ACEs had higher mental health scale means compared with 0–1 ACEs, except in the FPSS-Marital Stress subscale. Perceived Stress (PSS-4), PC-PTSD-5, GAD-7, and PHQ-8 were all significantly higher among those who experienced 2+ ACEs overall.

**Table 4.** Mental Health Variables and Adverse Childhood Experiences with Concurrent Reproductive Trauma (*n* = 59) and Infertility Only (*n* = 40) Groups

	ACE 0–1		ACE 2+			
Variable	*M*	SD	*M*	SD		
CRT (*n* = 59)	(*n* = 19)		(*n* = 40)		*t* (57)	*p*
FPSS—personal stress	11.21	1.58	11.93	1.21	1.92	0.060
FPSS—marital stress	8.16	1.80	8.70	2.45	0.86	0.394
FPSS—social stress	6.16	2.17	7.33	2.22	1.90	0.063
Perceived stress	7.63	2.31	8.75	2.71	1.55	0.127
PTSD	3.00	1.73	3.45	1.40	1.07	0.289
GAD	9.53	4.45	12.55	5.03	2.24	0.029
PHQ8	6.68	3.40	10.93	5.96	2.88	0.006

Infertility only (*n* = 40)	(*n* = 26)		(*n* = 14)		*t* (38)	
FPSS—personal stress	11.04	1.46	11.71	1.33	1.44	0.157
FPSS—marital stress	8.35	2.23	8.14	2.57	0.26	0.795
FPSS—social stress	6.31	2.38	6.71	2.76	0.49	0.629
Perceived stress	7.58	2.12	10.29	2.02	3.92	<0.001
PTSD	2.35	1.74	3.93	1.27	2.99	0.005
GAD	8.58	5.56	12.93	5.26	2.41	0.021
PHQ8	7.92	5.54	13.29	5.20	2.98	0.005

Note: CRT, concurrent reproductive trauma; FPSS, Fertility Problem Scale Score; GAD, Generalized Anxiety Disorder Scale; PHQ8, Patient Health Questionnaire; PTSD, post-traumatic stress disorder.

In Table 5, we compared clinically significant anxiety, depression, and PTSD scores between women who had a history of 0–1 ACEs and 2+ ACEs in each group (CRT vs infertility-only). The CRT group participants who experienced 2+ ACEs had clinically significant moderate/severe depressive symptoms compared with those who experienced 0–1 ACEs. Within the infertility-only group, the participants who experienced 2+ ACEs had clinically significant moderate/severe depressive and PTSD symptoms compared with those who experienced 0–1 ACEs.

**Table 5.** Mental Health Scale Clinical Significance Between Adverse Childhood Experiences among Concurrent Reproductive Trauma (*n* = 59) and Infertility Only (*n* = 40) Groups

	ACE 0–1		ACE 2+			
Variable	*n*	%	*n*	%		
CRT (*n* = 59)	(*n* = 19)		(*n* = 40)		**χ** ^2^	*p*
PHQ8						
Mild	14	73.7	16	40.0		
Mod/severe	5	26.3	24	60.0	5.85	0.016
GAD						
Mild	10	52.6	14	35.0		
Mod/severe	9	47.4	26	65.0	1.66	0.198
PTSD						
Unlikely	5	26.3	9	22.5		
Probable	14	73.7	31	77.5	0.10	0.748

Infertility only (*n* = 40)	(*n* = 26)		(*n* = 14)			
PHQ8						
Mild	18	69.2	4	28.6		
Mod/severe	8	30.8	10	71.4	6.08	0.014
GAD						
Mild	15	57.7	4	28.6		
Mod/severe	11	42.3	10	71.4	3.09	0.079
PTSD						
Unlikely	13	50.0	2	14.3		
Probable	13	50.0	12	85.7	4.95	0.026

Note: CRT, concurrent reproductive trauma; GAD, Generalized Anxiety Disorder Scale; PHQ8, Patient Health Questionnaire; PTSD, post-traumatic stress disorder.

## Discussion

This study aimed to explore the significance of ACEs in the context of reproductive traumas of infertility and pregnancy loss, which, to our knowledge, had not been previously studied. Our study found that women with CRT (pregnancy loss during infertility) had increased associations with having a history of experiencing specific ACEs, including neglect, substance use in the household, a household member incarcerated, and verbal abuse, when compared with women with infertility-only. We also found that while there were no significant psychological distress symptom differences between women with CRT and women with infertility-only, we found that there were differences between the two groups when we considered ACEs. Specifically, we found that women in both groups who had a history of 2+ ACEs reported worse mental health scores than women who did not experience any ACE or only 1 ACE. Our findings underscore the significant effect that ACEs have on the long-term mental health of women with infertility and how their compounding trauma experiences affect mental health outcomes.

Previous evidence suggests that women with a history of 3+ ACEs were almost two times more likely to have a miscarriage and nearly three times more likely to experience multiple miscarriages.^31^ In other studies, researchers found a dose-response relationship, signifying that each additional ACE increased the association to pregnancy loss.^21,22,32,33^ Mersky and Lee^21^ also found that 5± ACEs was associated with pregnancy loss in a study sample of 1,848 low-income mothers from Wisconsin. We found that women with infertility who had a history of 2+ ACEs were more likely to experience a miscarriage than those with 0–1 ACE. This may indicate that compounding traumas, such as childhood trauma and experiencing the trauma of infertility, could increase the risk of miscarriage. We also found that women with CRT reported a higher frequency of several ACEs compared with the infertility-only group, with significant associations involving neglect, substance use in the household, incarceration, and verbal abuse. This is consistent with other studies that found neglect and emotional abuse,^34,35^ substance use, and legal adversities in the household^33^ were associated with pregnancy loss. Demakakos et al.^31^ found an association between physical and sexual abuse and pregnancy loss; however, our study did not find that physical or sexual abuse was related to pregnancy loss among women with infertility. This could indicate that trauma experiences during childhood may affect women with infertility differently than women who do not experience reproductive diseases, although more studies are needed to investigate this further.

Although mental health scores were worse among the CRT group compared with the infertility-only group, it was not significantly different. This is a surprising finding, given that previous evidence suggested women who experience pregnancy loss also have worsening mental health symptoms such as depression, anxiety, and PTSD,^36^ and that infertility-related stress is worse among women experiencing CRT.^22^ The mental health outcomes of women with a history of ACEs who experience pregnancy loss are underreported in the literature. Stanhope et al.^37^ found that women who experienced ACEs and pregnancy loss were less likely to report the pregnancy loss as their most stressful life event when compared with women with pregnancy loss who did not experience ACEs. Shorter et al.^38^ found that ACEs were associated with depression and stress after miscarriage, but after controlling for race, the associations were not significant. These findings suggest that while women may appraise ACEs as more stressful than pregnancy loss, ACEs may not affect the development of depressive symptoms after pregnancy loss. In our study, the addition of ACEs in both groups of women with CRT and infertility-only affected the psychological distress outcomes, especially with depressive and anxiety symptoms, but they were worse among women with infertility-only. This suggests that the experience of ACEs may have a larger negative consequence on the psychological health of women with infertility and that women with CRT may have found additional support systems, such as social support, or other coping strategies that may have helped mitigate moderate to severe mental health disorders.

It is well known that mental health disorders increase in those who have a history of ACEs,^39^ and cumulative trauma exposure substantially increases the risk of poor mental health and physical health outcomes.^40^ Whitaker et al.^41^ reported that women were more likely to experience anxiety and depressive disorders with each ACE exposure, and a meta-analysis of 15 studies that included 7,788 women also found that women with a history of ACEs were more likely to experience depressive and anxiety symptoms during pregnancy and the postpartum period.^39^ Strathearn et al.^42^ found that women who experienced emotional abuse and neglect as children were more likely to have anxiety, depression, and PTSD at 21 years of age. These previous studies indicate that ACE experiences in women are associated with mental health disorders; however, these studies did not report mental health scores of participants or use the same measurements, thus we are unable to compare if mental health scores from our study of women with infertility differ from women in these other studies. Recurrent reproductive traumas, such as infertility and pregnancy loss, and the anticipation of continued infertility or future loss may contribute to cumulative psychological trauma.^12^ Cumulative trauma and chronic stress have been associated with neuroendocrine dysfunction, immune system dysregulation, systemic inflammation, and cardiovascular disease,^43^ which may partly explain the associations between ACEs and pregnancy loss, although further studies are needed.

### Clinical implications

Among the general population, almost 42% of women experience 2+ ACEs.^44^ Over half of the women in our study (55%) reported experiencing 2+ ACEs, reflecting the prevalence of childhood trauma in women with infertility and warrants future consideration. These women may be particularly susceptible to mental health complications, including moderate to severe anxiety, depression, and PTSD symptoms. Health care providers, including reproductive endocrinologists, OB-GYN physicians, nurse midwives, and nurse practitioners who care for women with infertility, could integrate ACE screening in their fertility treatment plans. This would allow providers to identify women who may be at higher risk for pregnancy loss and mental health complications, thereby facilitating individualized care plans that incorporate psychological support services. Olsen et al.^45^ reported that women were open to ACE screenings, and they found it helpful when providers explained the screening, had a calm and caring approach when screening, had support services available after the screening for emotional triggers, and provided education on the relevance of the screening to their health and well-being. Targeted interventions can be included in fertility care plans to decrease anxiety, depression, and stress among women with a history of ACEs facing reproductive difficulties. By recognizing the impact of ACEs on women with reproductive trauma, health care providers can better address the mental health needs of these women and improve their quality of care and psychological outcomes.

In addition, it is important to consider ACE history, as women may be retraumatized during fertility treatments or procedures. Feferkorn et al.^46^ found that women with a history of sexual abuse were triggered by some experiences during fertility treatments, such as securing hands or legs, transvaginal ultrasounds, or lying down on a procedure table. Patients can demonstrate trauma in other ways, such as anxiety, lack of eye contact, or distrust in providers and plans of care.^47^ Health care providers may implement trauma-informed approaches when caring for women with infertility and CRT, and especially for those with a history of ACEs. Trauma-informed care is a precautionary method of acknowledging patients with trauma and allowing providers to proactively build mutual trust and rapport with patients to reduce traumatization during health care visits. Trauma-informed care approaches include remaining at eye level, asking permission and providing clear explanations before making physical contact, and jointly creating a plan of care with patients.^47^ Including trauma-informed care practices may help improve patient satisfaction and mitigate the psychological distress of women with infertility, CRT, and a history of ACEs during health care visits for fertility treatments or procedures.

### Limitations

The cross-sectional design of this study limits causation among the variables. Furthermore, this study had a small sample size, and the participants were homogeneous with similar socioeconomic and demographic characteristics, which may limit the generalizability of findings. Future studies with larger, more diverse samples and longitudinal designs are recommended to validate associations between ACEs and CRT and to examine the enduring effects of ACEs and reproductive trauma on long-term mental health outcomes. This study relied on self-reported data, which may result in response bias by under- or overreporting their experiences based on their memory recall and social stigmas. We also did not ask participants about coping resources or strategies, which could have led to a deeper understanding of our findings.

## Conclusion

Our study found that women with CRT and infertility-only who experience two or more ACEs are at an increased risk for psychological distress. Women with CRT were also more likely to experience neglect, a substance-use household, a household member incarcerated, and verbal abuse at home than women with infertility-only. These findings indicate that ACEs are a potential risk factor for CRT and are associated with mental health disorders in women with infertility and CRT, though further studies are needed. Incorporating ACE screening and trauma-informed approaches within reproductive health care may help provide appropriate plans of care for women with infertility and CRT. Future research is needed to further understand the long-term implications of ACEs on reproductive diseases and trauma.

## Authorship Contribution

A.Swift.: Conceptualization, methodology, investigation, formal analysis, supervision, and funding acquisition. Writing—original draft. A.Schroeder.: Conceptualization, investigation, and writing—original draft. M.S.: Methodology, formal analysis, data curation, and writing—review and editing. M.F.-P.: Methodology, investigation, validation, and writing—review and editing.

## Abbreviations Used

ACE: adverse childhood experience
COMPI-FPSS: Copenhagen Multi-Centre Psychosocial Infertility Fertility Problem Scale Score
CRT: concurrent reproductive trauma
GAD: The Generalized Anxiety Disorder Scale
HIPAA: health insurance portability and accountability act
OB-GYN: Obstetrics-Gynecology
PC-PTSD: The Primary Care PTSD Screen for DSM-5
PHQ: The Patient Health Questionnaire
PSS: The Perceived Stress Scale
PTSD: post-traumatic stress disorder
QR: quick-response
SPARC: Sponsored Activities and Research Catalyst
SPSS: Statistical Package for Social Sciences
US: United States

## References

[1] Jaffe J. Reproductive trauma: Psychotherapy for pregnancy loss and infertility clients from a reproductive story perspective. Psychotherapy (Chic) 2017;54(4):380–385; doi: 10.1037/pst0000125 29251957

[2] Centers for Disease Control and Prevention. Reproductive Health. Infertility: Frequently Asked Questions. n.d. Available from: https://www.cdc.gov/reproductive-health/infertility-faq/index.html [Last accessed: July 23, 2025].

[3] Woods BM, Patrician PA, Fazeli PL, et al. Infertility‐related stress: A concept analysis. Nurs Forum 2022;57(3):437–445; doi: 10.1111/nuf.12683 34873709

[4] Bhat A, Byatt N. Infertility and perinatal loss: When the bough breaks. Curr Psychiatry Rep 2016;18(3):31; doi: 10.1007/s11920-016-0663-8 26847216 PMC4896304

[5] Choudhary G, Susmitha PP, Raju BJ. Comparative study of levels of anxiety, depression and quality of life between fertile vs infertile women attending a tertiary care center. Int J Psychiatry Res 2024;6(1):11–15; doi: 10.33545/26648962.2024.v6.i1a.60

[6] Öztürk R, Herbell K, Morton J, et al. “The worst time of my life”: Treatment‐related stress and unmet needs of women living with infertility. J Community Psychol 2021;49(5):1121–1133; doi: 10.1002/jcop.22527 33616236 PMC8324009

[7] Swift A, Reis P, Swanson M. Infertility-related stress and quality of life in women experiencing concurrent reproductive trauma. J Psychosom Obstet Gynaecol 2022;43(2):171–176; doi: 10.1080/0167482X.2021.2008901 34907847

[8] Wan F, Li A, Hao G, et al. Psychological status and influencing factors among pregnant women undergoing in vitro fertilization. Altern Ther Health Med 2023;29(6):393–399. 37442184

[9] Jackson T, Watkins E. Early pregnancy loss. Jaapa 2021;34(3):22–27; doi: 10.1097/01.JAA.0000733216.66078.ac 33528169

[10] Dar MA, Shah SB, Ahmad SN, et al. Psychiatric morbidity and quality of life in infertile females: A cross-sectional, case-controlled hospital-based study. Middle East Curr Psychiatry 2022;29(1); doi: 10.1186/s43045-022-00257-2

[11] Weng S, Chang J, Yeh M, et al. Do stillbirth, miscarriage, and termination of pregnancy increase risks of attempted and completed suicide within a year? A population‐based nested case–control study. BJOG 2018;125(8):983–990; doi: 10.1111/1471-0528.15105 29266732

[12] Brigance CA, Kim S-R, Kashubeck-West S. Mean comparisons of trauma symptoms between a reproductive trauma sample and a normative sample: Toward a trauma-informed practice. Psychol Trauma 2023;15(7):1164–1171; doi: 10.1037/tra0001468 36972102

[13] Hogg B, Gardoki-Souto I, Valiente-Gómez A, et al. Psychological trauma as a transdiagnostic risk factor for mental disorder: An umbrella meta-analysis. Eur Arch Psychiatry Clin Neurosci 2023;273(2):397–410; doi: 10.1007/s00406-022-01495-5 36208317

[14] Walters AJ, Notebaert L, Van Bockstaele B, et al. Occurrence of potentially traumatic events, type, and severity in undergraduate students. Aust Psychol 2025;60(1):43–53; doi: 10.1080/00050067.2024.2404983

[15] McKay MT, Cannon M, Chambers D, et al. Childhood trauma and adult mental disorder: A systematic review and meta‐analysis of longitudinal cohort studies. Acta Psychiatr Scand 2021;143(3):189–205; doi: 10.1111/acps.13268 33315268

[16] Daníelsdóttir HB, Aspelund T, Shen Q, et al. Adverse childhood experiences and adult mental health outcomes. JAMA Psychiatry 2024;81(6):586–594; doi: 10.1001/jamapsychiatry.2024.0039 38446452 PMC10918580

[17] Centers for Disease Control and Prevention. Adverse childhood experiences (ACEs). About adverse childhood experiences. n.d. Available from: https://www.cdc.gov/aces/about/index.html [Last accessed: May 30, 2025].

[18] Campbell JA, Walker RJ, Egede LE. Associations between adverse childhood experiences, high-risk behaviors, and morbidity in adulthood. Am J Prev Med 2016;50(3):344–352; doi: 10.1016/j.amepre.2015.07.022 26474668 PMC4762720

[19] Sanderson M, Mouton CP, Cook M, et al. Adverse childhood experiences and chronic disease risk in the Southern Community cohort study. J Health Care Poor Underserved 2021;32(3):1384–1402; doi: 10.1353/hpu.2021.0139 34421038 PMC8462987

[20] Swift A, Berry M, Fernandez‐Pineda M, et al. An integrative review of adverse childhood experiences and reproductive traumas of infertility and pregnancy loss. J Midwifery Womens Health 2024;69(2):258–278; doi: 10.1111/jmwh.13585 38013638

[21] Mersky JP, Lee CP. Adverse childhood experiences and poor birth outcomes in a diverse, low-income sample. BMC Pregnancy Childbirth 2019;19(1):387; doi: 10.1186/s12884-019-2560-8 31660899 PMC6819344

[22] Kerkar S, Shankar A, Boynton-Jarrett R, et al. Adverse childhood experiences are associated with miscarriage in adulthood: The GROWH study. Matern Child Health J 2021;25(3):479–486; doi: 10.1007/s10995-020-03079-y 33389588 PMC7965336

[23] Harris PA, Taylor R, Thielke R, et al. Research electronic data capture (REDCap)—A metadata-driven methodology and workflow process for providing translational research informatics support. J Biomed Inform 2009;42(2):377–381; doi: 10.1016/j.jbi.2008.08.010 18929686 PMC2700030

[24] Schmidt L. Infertility and assisted reproduction in Denmark. Epidemiology and psychosocial consequences. Dan Med Bull 2006;53(4):390–417. 17150146

[25] Sobral MP, Costa ME, Schmidt L, et al. COMPI fertility problem stress scales is a brief, valid and reliable tool for assessing stress in patients seeking treatment. Hum Reprod 2017;32(2):375–382; doi: 10.1093/humrep/dew315 27979919

[26] Cohen S. Perceived Stress in a Probability Sample of the United States. In: The Social Psychology of Health Sage Publications, Inc; 1988; pp. 31–67.

[27] Spitzer RL, Kroenke K, Williams JBW, et al. A brief measure for assessing generalized anxiety disorder: The GAD-7. Arch Intern Med 2006;166(10):1092–1097; doi: 10.1001/archinte.166.10.1092 16717171

[28] Kroenke K, Strine TW, Spitzer RL, et al. The PHQ-8 as a measure of current depression in the general population. J Affect Disord 2009;114(1–3):163–173; doi: 10.1016/j.jad.2008.06.026 18752852

[29] Prins A, Bovin MJ, Kimerling R, et al. Primary Care PTSD screen for DSM-5 (PC-PTSD-5) [Measurement Instrument] 2015. Available from: https://www.ptsd.va.gov

[30] ACEs Aware. Screening Tools. 2023. Available from: https://www.acesaware.org/learn-about-screening/screening-tools/

[31] Demakakos P, Linara-Demakakou E, Mishra GD. Adverse childhood experiences are associated with increased risk of miscarriage in a national population-based cohort study in England. Hum Reprod 2020;35(6):1451–1460; doi: 10.1093/humrep/deaa113 32510136 PMC7316498

[32] Hillis SD, Anda RF, Dube SR, et al. The Association between adverse childhood experiences and adolescent pregnancy, long-term psychosocial consequences, and fetal death. Pediatrics 2004;113(2):320–327; doi: 10.1542/peds.113.2.320 14754944

[33] Li Y, Margerison-Zilko C, Strutz KL, et al. Life course adversity and prior miscarriage in a pregnancy cohort. Womens Health Issues 2018;28(3):232–238; doi: 10.1016/j.whi.2018.02.001 29530382

[34] Abajobir AA, Kisely S, Williams G, et al. Risky sexual behaviors and pregnancy outcomes in young adulthood following substantiated childhood maltreatment: Findings from a Prospective Birth Cohort Study. J Sex Res 2018;55(1):106–119; doi: 10.1080/00224499.2017.1368975 28972390

[35] Freedman AA, Cammack AL, Temple JR, et al. Maternal exposure to childhood maltreatment and risk of stillbirth. Ann Epidemiol 2017;27(8):459–465.e2; doi: 10.1016/j.annepidem.2017.07.005 28755869 PMC5610090

[36] Westby CL, Erlandsen AR, Nilsen SA, et al. Depression, anxiety, PTSD, and OCD after stillbirth: A systematic review. BMC Pregnancy Childbirth 2021;21(1):782; doi: 10.1186/s12884-021-04254-x 34794395 PMC8600867

[37] Stanhope KK, Temple JR, Bann C, et al. Variation in self-identified most stressful life event by outcome of previous pregnancy in a population-based sample interviewed 6–36 months following delivery. Soc Sci Med 2021;282:114138; doi: 10.1016/j.socscimed.2021.114138 34153818 PMC8312027

[38] Shorter JM, Koelper N, Sonalkar S, et al. Racial disparities in mental health outcomes among women with early pregnancy loss. Obstet Gynecol 2021;137(1):156–163; doi: 10.1097/AOG.0000000000004212 33278280 PMC7737857

[39] Racine N, Devereaux C, Cooke JE, et al. Adverse childhood experiences and maternal anxiety and depression: A meta-analysis. BMC Psychiatry 2021;21(1):28; doi: 10.1186/s12888-020-03017-w 33430822 PMC7802164

[40] Fujiwara T. Impact of adverse childhood experience on physical and mental health: A life‐course epidemiology perspective. Psychiatry Clin Neurosci 2022;76(11):544–551; doi: 10.1111/pcn.13464 36002401

[41] Whitaker RC, Dearth-Wesley T, Herman AN, et al. The interaction of adverse childhood experiences and gender as risk factors for depression and anxiety disorders in US adults: A cross-sectional study. BMC Public Health 2021;21(1):2078; doi: 10.1186/s12889-021-12058-z 34772386 PMC8590371

[42] Strathearn L, Giannotti M, Mills R, et al. Long-term cognitive, psychological, and health outcomes associated with child abuse and neglect. Pediatrics 2020;146(4):e20200438; doi: 10.1542/peds.2020-0438 32943535 PMC7786831

[43] Dempster KS, O’Leary DD, MacNeil AJ, et al. Linking the hemodynamic consequences of adverse childhood experiences to an altered HPA axis and acute stress response. Brain Behav Immun 2021;93:254–263; doi: 10.1016/j.bbi.2020.12.018 33358983

[44] Swedo EA, Aslam MV, Dahlberg LL, et al. Prevalence of adverse childhood experiences among U.S. Adults — Behavioral risk factor surveillance system, 2011–2020. MMWR Morb Mortal Wkly Rep 2023;72(26):707–715; doi: 10.15585/mmwr.mm7226a2 37384554 PMC10328489

[45] Olsen JM, Galloway EG, Guthman PL. Exploring women’s perspectives on prenatal screening for adverse childhood experiences. Public Health Nurs 2021;38(6):997–1008; doi: 10.1111/phn.12956 34402097

[46] Feferkorn I, Goldberg A, Bonchek M, et al. Fertility treatment after sexual trauma. Reprod Biomed Online 2025;51(4):105022; doi: 10.1016/j.rbmo.2025.105022 40784289

[47] Grossman S, Cooper Z, Buxton H, et al. Trauma-informed care: Recognizing and resisting re-traumatization in health care. Trauma Surg Acute Care Open 2021;6(1):e000815; doi: 10.1136/tsaco-2021-000815 34993351 PMC8689164

